# Insights gained from early modelling of COVID-19 to inform the management of outbreaks in UK prisons

**DOI:** 10.1108/IJPH-09-2020-0075

**Published:** 2021-08-03

**Authors:** Declan Bays, Hannah Williams, Lorenzo Pellis, Jacob Curran-Sebastian, Oscar O'Mara, PHE Joint Modelling Team, Thomas Finnie

**Affiliations:** Emergency Response Department, Public Health England, London, UK; Department of Mathematics, The University of Manchester, Manchester, UK; Her Majesty's Prison and Probation Service, London, UK; Public Health England, London, UK; Emergency Response Department, Public Health England, London, UK

**Keywords:** Health in prison, Modelling, Health policy, Infectious disease, Prisoner health, Covid-19

## Abstract

**Purpose:**

In this work, the authors present some of the key results found during early efforts to model the COVID-19 outbreak inside a UK prison. In particular, this study describes outputs from an idealised disease model that simulates the dynamics of a COVID-19 outbreak in a prison setting when varying levels of social interventions are in place, and a Monte Carlo-based model that assesses the reduction in risk of case importation, resulting from a process that requires incoming prisoners to undergo a period of self-isolation prior to admission into the general prison population.

**Design/methodology/approach:**

Prisons, typically containing large populations confined in a small space with high degrees of mixing, have long been known to be especially susceptible to disease outbreaks. In an attempt to meet rising pressures from the emerging COVID-19 situation in early 2020, modellers for Public Health England’s Joint Modelling Cell were asked to produce some rapid response work that sought to inform the approaches that Her Majesty’s Prison and Probation Service (HMPPS) might take to reduce the risk of case importation and sustained transmission in prison environments.

**Findings:**

Key results show that deploying social interventions has the potential to considerably reduce the total number of infections, while such actions could also reduce the probability that an initial infection will propagate into a prison-wide outbreak. For example, modelling showed that a 50% reduction in the risk of transmission (compared to an unmitigated outbreak) could deliver a 98% decrease in total number of cases, while this reduction could also result in 86.8% of outbreaks subsiding before more than five persons have become infected. Furthermore, this study also found that requiring new arrivals to self-isolate for 10 and 14 days prior to admission could detect up to 98% and 99% of incoming infections, respectively.

**Research limitations/implications:**

In this paper we have presented models which allow for the studying of COVID-19 in a prison scenario, while also allowing for the assessment of proposed social interventions. By publishing these works, the authors hope these methods might aid in the management of prisoners across additional scenarios and even during subsequent disease outbreaks. Such methods as described may also be readily applied use in other closed community settings.

**Originality/value:**

These works went towards informing HMPPS on the impacts that the described strategies might have during COVID-19 outbreaks inside UK prisons. The works described herein are readily amendable to the study of a range of addition outbreak scenarios. There is also room for these methods to be further developed and built upon which the timeliness of the original project did not permit.

## Introduction

On 5 February 2020, the cruise ship Diamond Princess was quarantined in the port of Yokohama, Japan, following a series of passengers (past and present) being positively diagnosed with COVID-19. A two-week lockdown was subsequently imposed, which saw passengers being quarantined inside their cabins for the duration to minimise the risk of further spread (National Institute of Infectious Diseases, 2020). Within two weeks, an additional 619 passengers and crew members (16.7% of the total onboard population) were positively identified as being infected with COVID-19 (Dahl, 2020). In light of these events, concerns were raised about the severity of possible outbreaks should COVID-19 enter into similarly confined populations, or “closed communities” (Koshkouei *et al.*, 2020; WHO, 2020), which have historically been susceptible to outbreaks of influenza-like infectious diseases (Finnie *et al.*, 2012; Finnie *et al.*, 2014; Lansbury *et al.*, 2017).

Prisons and detention centres have long been known to be particularly vulnerable to epidemics (Young *et al.*, 2005; Reid *et al.*, 2012). This is predominantly due to the fact that such institutions are typically densely populated, with incarcerated populations living within close proximity with high degrees of mixing. High turnover rates and an elevated rate of respiratory illness, immunosuppression and other chronic disease among prisoners are other key contributing factors (Public Health England, 2019).

As the COVID-19 situation started to quickly develop in the UK, Her Majesty’s Prison and Probation Service (HMPPS) made a request for rapid response analytical work from Public Health England’s Joint Modelling Cell, for the purposes of developing evidence-based and informed policy that focused on reducing the risk of importation and minimising the potential for sustained transmission within a prison. The subsequent works considered the impact that proposed social interventions (such as social distancing, isolating symptomatic cases and shielding extremely vulnerable prisoners) might have upon outbreaks in prison settings. Also investigated was the affect that requiring all incoming prisoners to undergo a period of isolation prior to release into the general prison community might have on unknowingly releasing infection into the wider population.

In this paper, we present a brief overview of the models used and present some of the key findings that were obtained during these efforts. However, due to the rate at which the COVID-19 epidemic was unfolding, the models that were developed for this work considered only the key features that were of primary interest and did not take into consideration non-COVID-19 related health impacts, such as prisoner welfare. As such, the results we present here should be interpreted as broad stroke indicators rather than absolute predictions. It is hoped that the dissemination of the included results might better inform decisions that are made on the handling of prisoners during the COVID-19 epidemic. Moreover, with the flexibility in the methods presented, we believe the approaches described herein may also find applicability when modelling other communicable disease outbreaks in similarly structured closed community settings.

## Methods

This paper is a compilation of various completed works with multiple models having been used to obtain our reported results. We present an idealised in-prison disease model (which we evaluate both deterministically and stochastically) that has been used to simulate the dynamics of an outbreak occurring in a prison setting, and another model that uses Monte Carlo simulation to estimate the impact that requiring new prisoners to undergo a period of self-isolation might have on the probability of case importation. We present an overview of each of these models in turn below.

### Terminology

Within this work we have assumed the definition of several terms:

- “**Clinically attacked**” is taken to be a person who is identified as infected through the display of symptoms following incubation. Such persons will then be isolated to prevent mixing during the remainder of their infectious period. We assume the remainder of the infected population that are not “clinically attacked” are either asymptomatic or successfully hide their symptoms.
- “**Cohorting**” to mean the removal of clinically attacked cases from the general prison population, so that they are not able to infect anyone else.
- **“Reverse cohorting”** is the process of requiring incoming prisoners (new arrivals) to undergo a period of self-isolation prior to admittance into the general prison population. Prisoners undergo diagnostic testing (which we assume returns a positive result should prisoners have completed their incubation period by the time testing is administered) upon entry into, and transfer from, the **“reverse cohorting unit (RCU)”**.
- “**Shielding**” as the removal of extremely clinically vulnerable prisoners from the general prison population. While being shielded, such prisoners are assumed to be fully isolated from risk of infection.

### In-prison disease model parameterisations and assumptions

We present some of the key assumptions as made for the disease model in the following work (for a full list of assumptions, please see the Appendix). As in-depth literature was sparse at the time of initial development, many of these assumptions were provided by HMPPS (informed through their own data) to give best estimates of the disease dynamics in the scenarios being considered:

- Each model run starts with a single infected prisoner that is incubating – this seeding event is assumed to be spontaneous.
- The prison population is assumed to be homogeneous and mix uniformly, disregarding strict confinement between blocks or wings.
- Contact between prisoners and prison staff is assumed to be negligible in comparison to the number of contacts with other prisoners. As such, the effect of prison staff on the dynamics of an outbreak is not considered.
- There is a 50% probability that infected prisoners shall be clinically attacked.
- Infected prisoners that are not detected by prison staff recover (i.e. are no longer infectious) on average after 4 days from the start of infectiousness.
- The model is parameterised to achieve an R_0_ value of 3.0 in the unmitigated scenario to conform with reported values of the reproductive number at the height of the UK’s first wave (Mahase, 2020) (we note that this value is representative of the reproduction rate in the general public, not a closed community setting, for which no data was available).
- It takes on average 1 day for clinically attacked prisoners to be detected after the completion of their incubation period.
- For infected prisoners that are detected, it takes on average 7 days for clinically attacked prisoners to fully recovery if suffering from mild symptoms, and on average 22 days for clinically attacked prisoners that have been hospitalised to fully recover.
- Infected prisoners take on average 6 days to incubate.
- Infected prisoners become infectious upon completion of their incubation period.
- A prison contains 678 prisoners; obtained by averaging prison data as reported in August 2020 (Ministry of Justice, 2020).
- In accordance with average data provided by HMPPS, 1.2% of the prison population are designated as extremely clinically vulnerable.

### In-prison disease model

**Figure 1:**
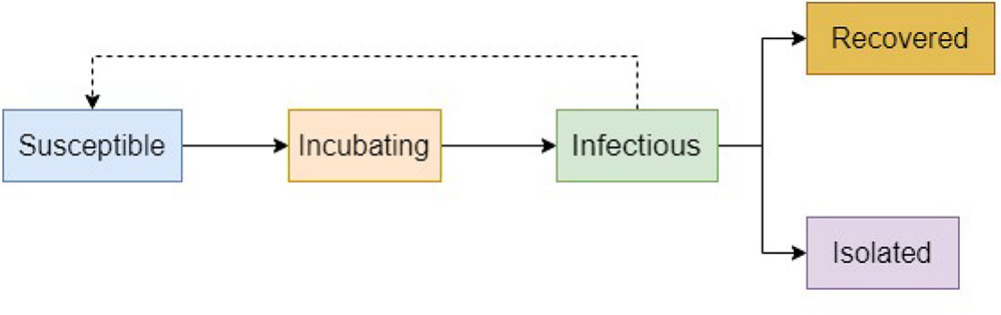
Flow diagram outlining key features of the disease progression implemented by the in-prison model.

The model that we have elected to use to simulate disease dynamics in a prison setting is based upon a compartmental model that considers the distinct stages of a COVID-19 infection and allows for the isolation of identified cases. This model was chosen as our framework as it provided the best fit to published COVID-19 case and death data; our model was then extended to better capture the disease dynamics as observed in the COVID-19 outbreak in the population on board the Diamond Princess following the enforcement of quarantine (passengers being confined in their own cabins for a two week period (National Institute of Infectious Diseases, 2020)) – a scenario that we consider to be comparable to an outbreak inside a prison. The flow diagram presented below gives a simple overview of how disease progression is governed in the modelled prison population (for a more detailed description of this model, please see the Appendix) (Figure 1).

The rates at which individuals in any of these distinct states progress to subsequent states is determined according to the parameters given in our assumptions. In particular, the rates at which susceptible individuals’ transit into the incubating state (i.e. are infected), and those in the infectious state transit into the recovered state are driven by the *force of infection* (typically represented by β) and the average time that individuals remain infectious respectively (in the Appendix, which contains explicit descriptions of these relationships, we denote this value by t_g_). These values also define R_0_ according to the product R_0_ = *β*·t_g_. With our given assumptions (R_0_ = 3.0, t_g_ = 4.0), this gives our base value of *β* = 0.75 during an unmitigated outbreak. However, we note that the force of infection encapsulates high level information about the system being considered, such as the general infectivity of the given disease and the social structure of the population (e.g. average number of daily contacts between members of the given population). For this reason, the R_0_ value for a given disease is not fixed and can range across population structures. We make this point to highlight that our assumed value of R_0_ may vary from the true value for COVID-19 in closed community settings. Nevertheless, as mentioned in the assumptions section, this value was adopted in lieu of further evidence.

While our model takes account of the isolation of identified infections through the inclusion of an “isolated” pathway in the disease progression (as depicted in our flow diagram), to capture the effects of social interventions of differing severities we introduce a scaling on the force of infection. For reasons given in the above paragraph, we can assume that β may be used as a proxy measure for the general risk of infection. Therefore, we model the introduction of varyingly strict social interventions by reducing the value of the force of infection to 75%, 50% and 25% of its base value. Due to the relationship described above, one may assume that such reductions may roughly correlate to proportional decreases in the average number of social contacts each prisoner has per day. Thus, the values of 75%, 50% and 25% then represent varying levels of social distancing stringency/compliance. Steps taken to reduce the general infectivity of the disease (i.e. risk of infection given contact with infected persons), such as by improving hand hygiene and enforcing mask wearing, may also deliver such reductions. We subsequently use the terms “reduction in force of infection” to refer to the implementation of social interventions and vice versa.

We also use this model to assess what impact shielding the proportion of the prison population deemed to be “extremely clinically vulnerable” to severe infection would have on the dynamics of the overall outbreak. However, as the effectiveness of shielding in the real-world is not easily quantifiable, we make the proxy of assuming it is 100% effective; when shielding is being enforced, the highly vulnerable population are assumed to be completely isolated so their numbers are simply removed from the starting prison population.

We clarify that this model was not intended to provide a precise depiction of COVID-19 outbreaks in a prison setting, but rather to inform scenario planning in the absence of detailed information on the COVID-19 virus and its behaviour in closed community settings. As such, we have neglected some components of a typical prison structure that we have assumed (mainly due to lack of confirmatory information) would not significantly impact the dynamics of an outbreak once there has already been ingress (such as the effect of prison staff; given the relatively small number of expected staff-to-prisoner contacts in comparison to prisoner-to-prisoner contacts, we assumed prisoner-to-prisoner contacts to be the main driving force behind the spread of infection in the prisoner population).

### Computation of disease model

For the construction and computation of our in-prison model we have elected to use the Python-based ordinary differential equation modelling package Python General ODE Modelling (PyGOM) (Public Health England, 2020a). This package allows for the running of both deterministic and stochastic models. We start by implementing our model using PyGOM’s deterministic functionality. Through this we model mitigated (where the isolation of detected cases and shielding of the vulnerable population is being enforced) and unmitigated outbreaks while reductions to the force of infection is also applied. We then run the model stochastically (10,000 times for each scenario), which better considers the random nature of events involved in true disease progression (such as the processes which determine whether infectious prisoners will be detected or not, or whether those that are detected will have a mild or severe infection). Running the model stochastically also allows us to capture fringe real-world events that might not be observable through deterministic modelling, such as stochastic die-out. The stochastic simulations presented below only consider outbreaks in a prison already implementing mitigating interventions. As such, the results presented only vary according to the reduction imposed on the force of infection.

A Jupyter Notebook containing the code used to compute this model has been provided in a publicly accessible GitHub repository (Public Health England, 2021).

### Reverse cohorting model parameterisation and assumptions

We present some of the key assumptions made for the reverse cohorting model in the following:

- All simulated prisoners have, at some point prior to entering the RCU, been infected with COVID-19.
- Incubation time is sampled according to a log-normal distribution with the parameters.
- *µ* = 1.6112, *σ* = 0.47238. These values were taken from published literature(Linton *et al.*, 2020).
- For each prisoner, the time of infection is sampled according to uniform distribution over the 14 days prior to entering the RCU.
- This model does not consider disease recovery (once a prisoner becomes detectable, they remain so “indefinitely”).
- Prisoners are only tested upon entry into the RCU, and again after they have spent the specified amount of time in the RCU (if they go undetected by the first test).
- Results from testing are assumed to be returned instantaneously.
- If a prisoner has become detectable prior to a test, a weighted coin-toss (with probability of success equal to the testing sensitivity) is used to decide whether their next administered test identifies their infection.
- Infection is only detected through testing. In particular, we do not consider the case where infection is identified through symptomologies.
- All prisoners undergo the reverse cohorting process.
- There is no disease transmission at any stage of the model.
- Simulated individuals proceed through the model one at a time, and thus enter self-isolation alone.

### Reverse cohorting model

To calculate the expected probabilities that an infected prisoner will be go undetected having spent a given period in an RCU, we have adapted a Monte Carlo-based model that was developed by the authors for other work (Bays *et al.*, 2020). This model incorporates known aspects of COVID-19’s epidemiology, and simulates prisoners being infected at some randomly sampled time prior to attempting to enter the RCU at time *t* = 0. According to each simulated prisoner’s time of infection and incubation period, prisoners are then assessed to determine whether they would be detected by testing that is administered upon entry into, and transfer from, the RCU. If detected at any point, prisoners are removed from the model and recorded as detections. This is repeated 100,000 times to evaluate a single scenario. We then repeat this process for each of the varying lengths of time spent in the RCU and testing sensitivities being considered. The output of this model then approximates the probability that a prisoner who has been infected prior to arrival would successfully be admitted into the wider prison population (i.e. remain undetected) under each of the given scenarios and as such we do not need to consider the inclusion of uninfected individuals. A full break down of the algorithm employed by this model is provided in the Appendix.

### Computation of reverse cohorting model

To compute this model, we have used the python package simulated importation risk assessment (Public Health England, 2020b) in conjunction with the parameters described above.

A Jupyter Notebook containing the code used to compute this model has been provided in a publicly accessible GitHub repository (Public Health England, 2021).

## Results

### Results from deterministic in-prison disease model

We present results obtained from a deterministic implementation of our disease model that considers mitigated (where cohorting and shielding of vulnerable prisoners is being enforced) and unmitigated scenarios that also incorporate 0%, 25%, 50% and 75% reductions in force of infection.

**Figure 2:**
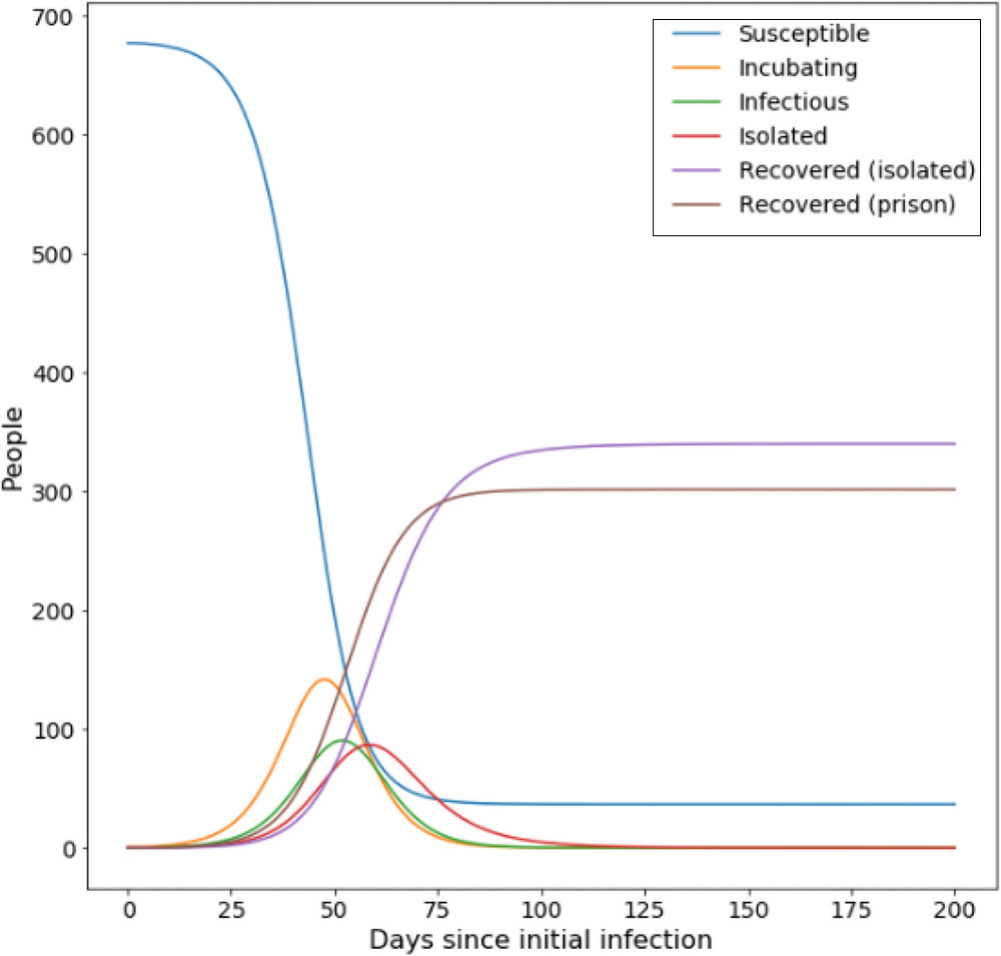
Evolution of disease states during an unmitigated outbreak in an average prison, while no transmission risk reducing steps are in place

We start by considering an outbreak occurring where no intervening steps are being enforced (no cohorting, no shielding and a 0% reduction in force of infection) (Figure 2).

This outbreak results in a clinical attack rate of 50.13%, with 94.59% of the total population being infected. Of those infected, 5.83% end up hospitalised with severe infection (5.51% of the total population). Peak number of infections occurs 52 days after initial introduction.

**Figure 3:**
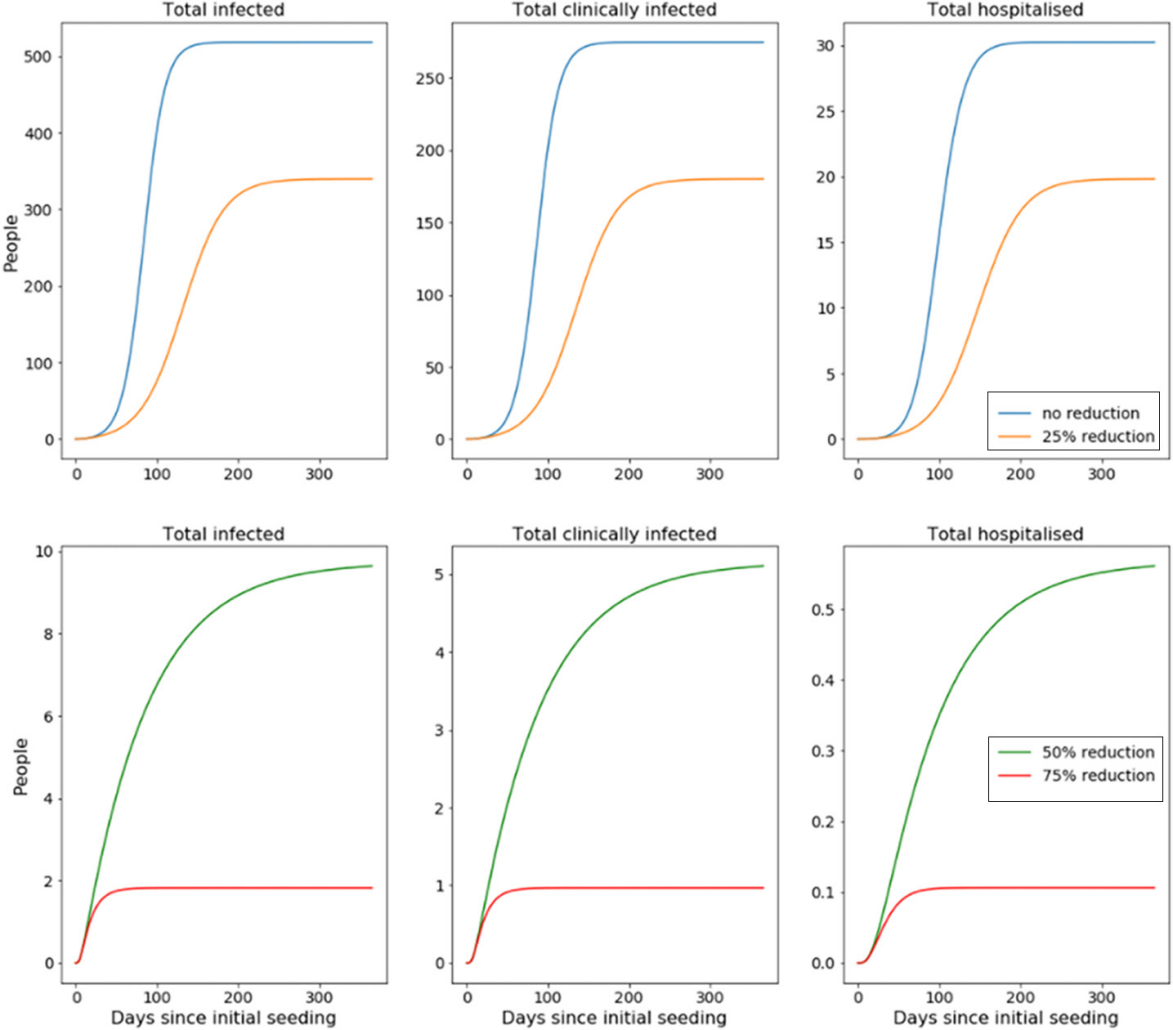
Evolution of cumulative number of total infected (detected and undetected) and hospitalised prisoners compared for outbreaks mitigated with varying levels of reduction in transmission risk

Next, we look to compare this against similar outbreaks where mitigating interventions are in place (cohorting and shielding are being enforced), while reductions to the risk of transmission are also being applied (Figure 3).

We also ran our model where a reduction to the force of transmission is the only intervention being considered (no shielding or cohorting). Both sets of results are compiled in the table below (Table 1).

### Results from stochastic in-prison disease model

**Figure 4:**
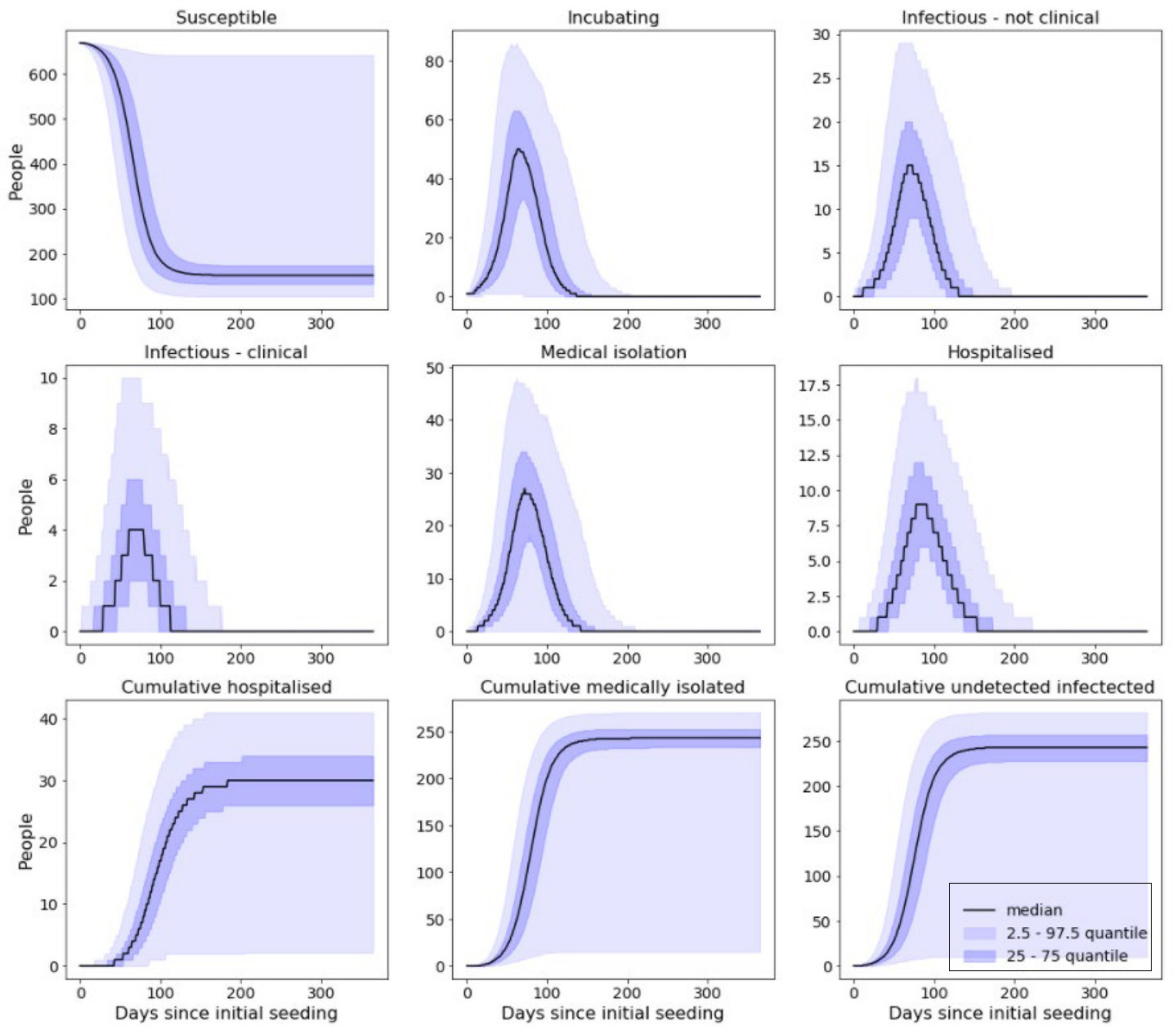
Model output from simulated prison with no reduction in force of infection

For each of the following scenarios, results presented have been compiled from 10,000 individual simulations. All of the scenarios presented consider the shielding of vulnerable prisoners and cohorting of detected cases, but vary by reductions in force of infection. Simulated outbreaks that resulted in less than five cases were removed from these presented results; rates at which five or fewer cases occurred for each scenario are presented at the end of this section. (Figure 4)

With shielding and cohorting in place but no reduction in force of infection, over the course of the outbreak we would expect to see approximately:

- 76.1% of a prison population become infected.
- 40.2% of a prison population be clinically attacked.
- 4.4% of a prison population requiring hospitalisation.

**Figure 5:**
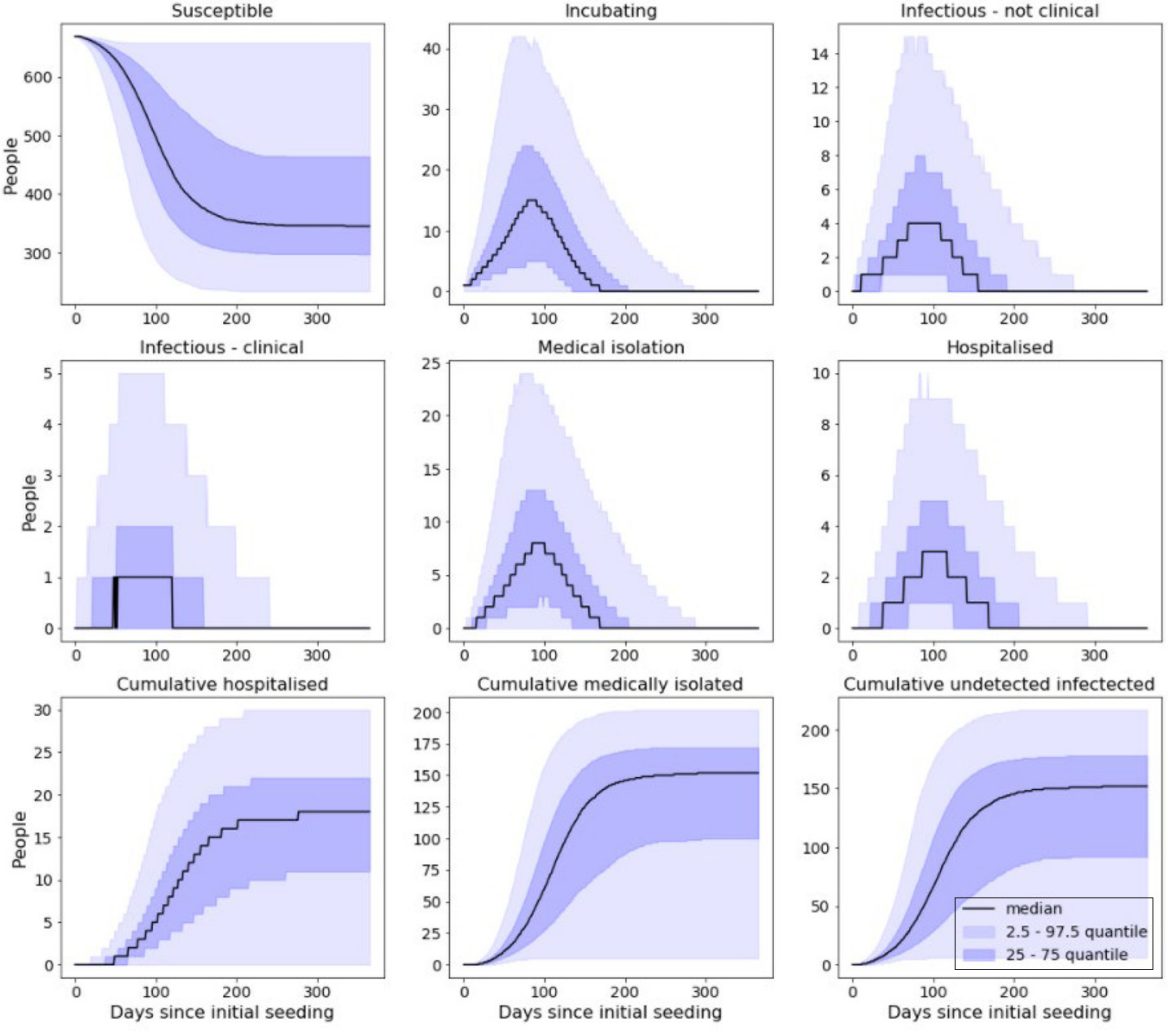
Model output from simulated prison with 25% reduction in force of infection

With shielding and cohorting in place and a reduction of 25% in force of infection, over the course of the outbreak we would expect to see approximately (Figure 5):

- 47.5% of a prison population become infected.
- 25.1% of a prison population be clinically attacked.
- 2.7% of a prison population requiring hospitalisation.

**Figure 6:**
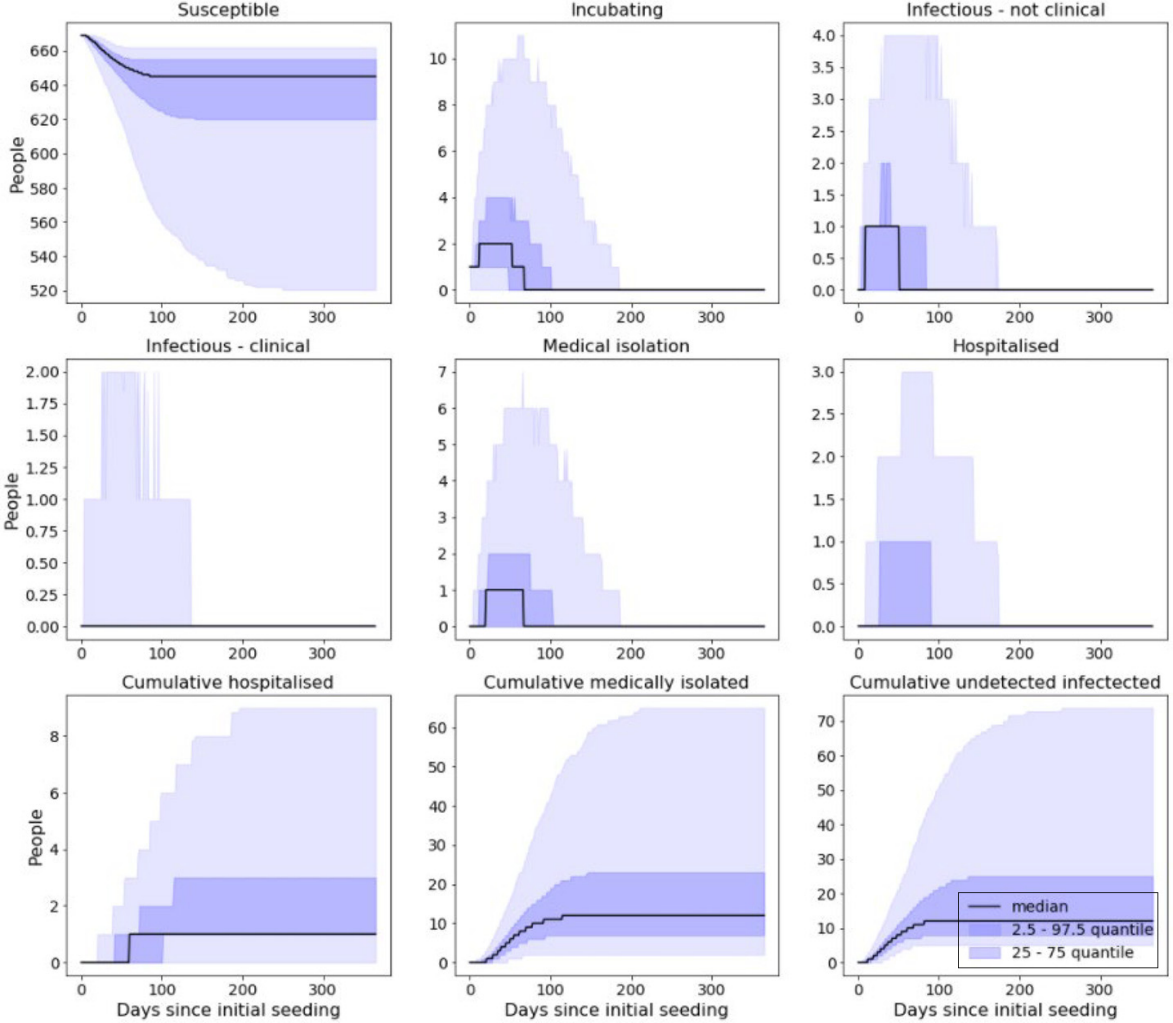
Model output from simulated prison with 50% reduction in force of infection

With shielding and cohorting in place and a reduction of 50% in force of infection, over the course of the outbreak, we would expect to see approximately (Figure 6):

- 3.7% of a prison population become infected.
- 1.9% of a prison population be clinically attacked.
- 0.1% of a prison population requiring hospitalisation.

**Figure 7:**
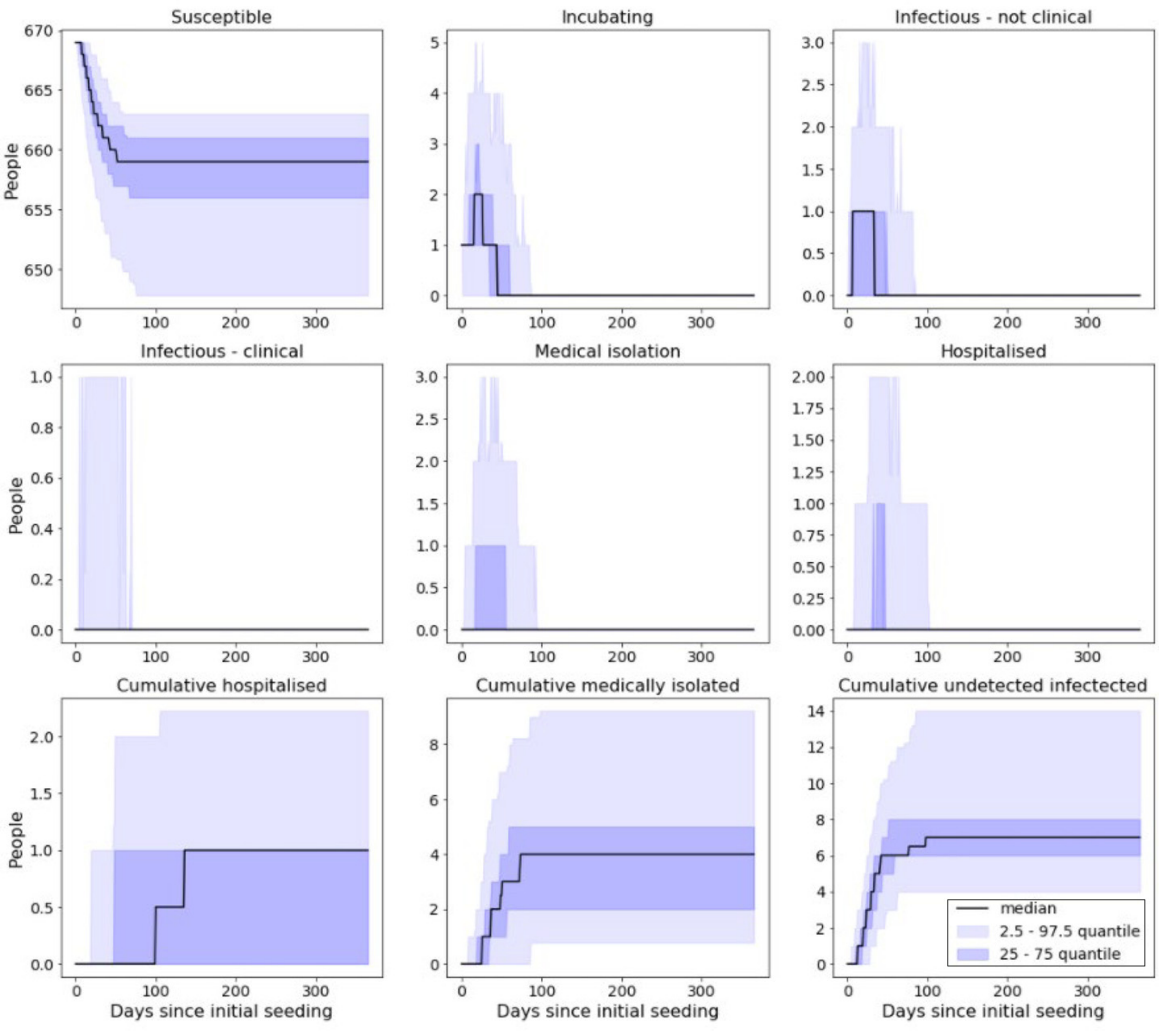
Model output from simulated with 75% reduction in force of infection

With shielding and cohorting in place and a reduction of 75% reduction in force of infection over the course of the outbreak, we would expect to see approximately (Figure 7):

- 0.3% of a prison population become infected.
- 0.1% of a prison population be clinically attacked.
- 0.0% of a prison population requiring hospitalisation.

We have collated all the above results into the below table for easy comparison (Table 2).

### Effect of reducing force of infection on the chance of stochastic die-out

In addition to reducing the total number of infections during an outbreak, reducing the force of infection can also be expected to increase the likelihood that outbreaks will prematurely abate due to stochastic die-out. This is due to the decrease in the likelihood of sustained transmission. Rates at which the above simulated outbreaks resulted in less than five total infections are presented in this table (Table 3).

### Results from modelling the reverse cohorting process

The table presented below provides the probability that an incoming prisoner, having been infected prior to arrival at prison, remains undetected after spending a given amount of time isolated in the RCU when a test of given sensitivity is being deployed (on both arrival and departure from RCU) (Table 4).

Note that the values reported in the row “None” represent the probability that infected prisoners will either still be undetectable upon entry into the RCU, or that have become detectable prior to entering the RCU but will not be detected by the test administered upon entry due to the false negative rate of the given test. Equivalently, these can also be interpreted as the probability that an infected prisoner will not be detected prior to admittance to the general prison population when incoming prisoners are only tested on arrival and reverse cohorting is not being deployed.

## Discussion

We start by considering the observed impact that followed from cohorting clinically attacked prisoners. From the outputs of the deterministic implementation of our disease model, shown in Figure 5, it can be seen that the action of shielding extremely clinically vulnerable prisoners and cohorting detected infections has a considerable effect. Data shows that by taking these steps alone, we may expect total number of infections to reduce by 19.2% and the time of peak number of infections to be delayed by 53%. However, if we also consider a reduction in force of infection, the results are starker; taken in conjunction with a 25% reduction in the force of infection, we see a 42% decline in the total number of infections as well as a delay in the time of peak number of infections by 91% – compared to a 9% decrease in total number of infections and a 29% delay in time of peak number of infections when a 25% reduction in force of infection is implemented alone (without shielding and cohorting). While we parameterised the model so that the base unmitigated scenario had an R_0_ of 3.0, the introduction of cohorting and shielding reduces this down to 1.81 without any further action. This explains why combining shielding and cohorting with a 50% or more reduction in the force of infection results in an outbreak failing to arise from our index case (as this then brings R_0_ below 1 – recalling R_0_ = *β*·t_g_, where *β* represents the force of infection). Failure for outbreaks to spread is also seen to occur in scenarios where cohorting and shielding is not enforced, but not until a larger reduction in risk of transmission is introduced (so that the unmitigated R_0_ of 3.0 is scaled to 1.0 or less).

Next, we look at the effect that reducing the force of infection has upon the final size of an outbreak. As is apparent from the results of all our disease models, reducing force of infection acts to reduce the total size of the outbreak while also delaying the time of peak number of infections. This is because, by taking steps to reduce the force of infection, we are essentially decreasing the likelihood of subsequent infections following from each infectious case. In the stochastic model, this also increases the probability that cases recover before being able to spread their infection. Our deterministic model showed that a reduction of 50% in the force of infection alone lead to a reduction of 38% in the total number of prisoners infected, with this reduction spiking to 98% when the implementation of cohorting and shielding is also being enforced. A 75% reduction in force of infection resulted in a reduction of 99% in the total number of infections across both scenarios. The effects of reducing the risk of transmission were even more evident in the stochastic implementations of our model; 25% and 50% reduction in the force of infection resulted in 33% and 98% fewer total cases, respectively (however, recall that the stochastic model only considered the scenarios where shielding and cohorting were being enforced).

Our results also indicated that reducing force of infection also lead to a reduction in the rate at which the outbreak spreads, which could ultimately allow prison staff more time to prevent subsequent cases. In the results from our deterministic model, we can see that in the non-cohorting scenarios, 25% and 50% reductions in the force of infection resulted in the time of peak number of infections moving from 52 days to 67 and 113 days after the introduction of the initial case respectively. While in the cohorting scenarios, time of peak number of infections came 80 and 128 days after the initial introduction for 0% and 25% reductions in force of infection.

This effect is mirrored in the stochastic simulation that considered a 25% reduction. However, for our other sets of results the effects of the respective reductions reduce the chances of an outbreak propagating to such a point that it does not build up enough momentum to result in more than three consecutive cases. As such, these peaks occur very soon after the initial introduction and die off quickly thereafter. As shown in Table 3, the introduction of interventions also acts to increase the probability of stochastic die-out; all active cases being either removed from the wider population or managing to recover before they can seed any additional cases. Notably, as seen in Table 3, this model showed that by just shielding the extremely clinically vulnerable prisoners and cohorting identifiably symptomatic infections (0% reduction in force of infection – Figure 4), we can expect over half of outbreaks to not result in more than five cases. These effects become more pronounced from then on, with 50% and 75% reductions in the force of infection result in over 86% and 98% of introductions resulting in less than five total infections.

Finally, we discuss the results from our reverse cohorting model. The most immediate result from this work is that the enforcing of reverse cohorting of any kind (regardless of how poor the testing sensitivity or time spent in the RCU) reduces the risk of case importation substantially. We see that in the worst considered case (spending 5 days in RCU with testing sensitivity of 50%), reverse cohorting can be expected to detect over 60% of incoming cases. However, current evidence suggests that tests being used in the UK have a sensitivity of over 90% (Burki, 2020). Thus, for the same five days of isolation, we might expect that the reverse cohorting process would be able to detect over 86% of incoming cases. Extending the time of isolation to 14 days (as is currently required for persons to self-isolate in the UK), this rate of detection jumps to over 95%. Further reports indicate that testing might be closer to 100% effective at detecting true positives (ECDC, 2020), and hence isolating individuals for 10 or 14 days would be expected to detect over 98% and 99% of all incoming infections, respectively.

## Conclusion

In this paper we have reconstructed models which were produced to provide early insights into the possible COVID-19 situation in UK prisons. These included a model that considered the in-prison disease dynamics of a COVID-19 outbreak and a model that simulated the process of isolating new prison arrivals prior to releasing them into the prison population. We then evaluated these models under varying situations to investigate how an outbreak might evolve in a prison setting, as well as what protection isolating all prison arrivals might give from importing COVID-19 cases into the wider population. In particular, our in-prison model considered situations where prisons are or are not isolating extremely clinically vulnerable prisoners and clinically attacked prisoners, while also enforcing policies that aim to reduce the risk of disease transmission by varying amounts. Results from this model showed that by isolating clinically attacked prisoners, we can reduce the size of an outbreak and delay the time in which it peaks quite substantially (by 19% and 42%, respectively) without considering any further interventions. Furthermore, if we use this cohorting method in conjunction with reducing the risk of transmission, the effects are amplified; cohorting, shielding and reducing force of infection by 50% result in only 1.4% of the total population being infected. When cohorting and shielding is not enforced, a reduction of 75% in the force of infection is required to obtain a result comparable. Additionally, we used a Monte Carlo-based model to evaluate the reverse cohorting procedure that requires new prison arrivals to isolate prior to being received into the general prison population. This model simulates prisoners that have been infected at some random time prior to arrival at the prison, entering into the RCU (so long as they are not detectable by this point and subsequently picked up by testing on entry) and spending a given amount of time self-isolating before being discharged into the prison (if not detected by a test administered upon completion of the self-isolation period). Results showed that requiring incoming prisoners to undergo reverse cohorting for any given period increases the likelihood that infections will be detected prior to transfer into the general prison. Results also showed that implementing this process has the potential to detect 98% and 99% should all arrivals be isolated for 10 and 14 days, respectively. These results went towards informing HMPPS of the impact that the described strategies might have during COVID-19 outbreaks (O’Moore, 2020).

Beyond the scope of this paper, the methods presented here remain flexible. The in-prison model described may be easily amended and applied to model arbitrary communicable respiratory disease in other closed communities, while the reverse cohorting model has already been applied in other settings (Bays, Bennett and Finnie, 2020). By publishing these methods, we hope that they might be properly scrutinised and developed s to produce an improved toolset for the management of prisoners (or other confined populations) during subsequent disease outbreaks.

## Figures and Tables

**Figure A1:**
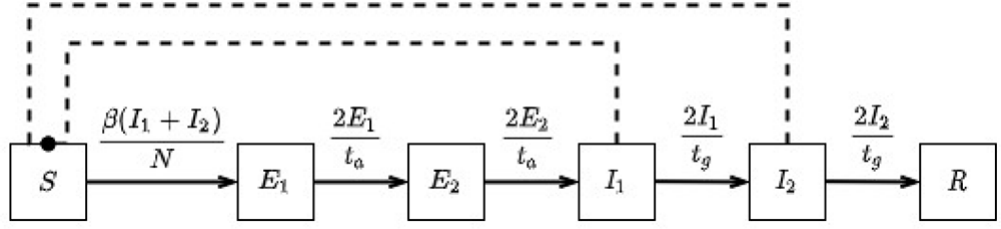
Flow diagram displaying our basic in-prison disease model (with equations governing transitions) comprised of states: S – susceptible, E – exposed, I – infectious, R – recovered

**Figure A2:**
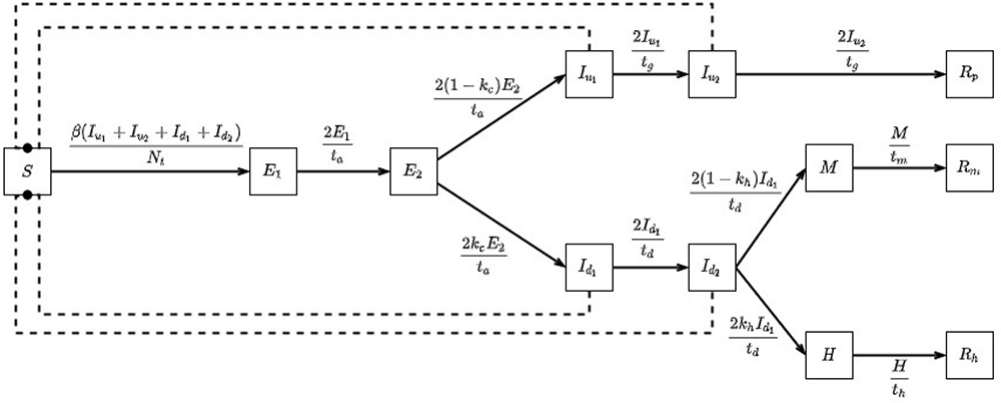
Flow diagram displaying our extended in-prison model (with equations governing transitions) comprised of states: S – susceptible, E – exposed, I_u_ – infectious (will not be detected), I_d_ – infectious (will be detected), M – medically isolated, H – hospitalised, R – recovered (with subscripts *p*, m and h representing those in the main prison population, medical isolation and hospital respectively). Notice that by setting the parameter kc to the value 0, this model reduces to our basic model

**Table 1:** Results obtained from deterministic implementation of disease model that considers the effect of reducing force of infection with and without the shielding of extremely clinically vulnerable and cohorting of clinically attacked prisoners

Reduction in force of infection (%)	Shielding and cohorting	Clinical attacked (%)	Total infected (%)	Hospitalised (%)	Time of infection peak (days after introduction)
0		50.13	94.59	5.51	52
0	✓	41.01	77.37	4.51	80
25		45.78	86.38	5.03	67
25	✓	26.88	50.72	2.96	128
50		31.07	58.92	3.31	113
50	✓	0.76	1.44	0.08	8
75		0.31	0.58	0.03	8
75	✓	0.14	0.27	0.02	6

**Table 2:** Combined outputs from stochastic model showing the impact that various reductions in force of infection have on infection and hospitalisation rates

Reduction in force of infection (%)	Total infected (%)	Total clinically attacked (%)	Total hospitalised (%)
0	76.10	40.20	4.40
25	47.50	25.10	2.70
50	3.70	1.90	0.10
75	0.30	0.10	0.00

**Table 3:** Probabilities that a single introduction will result in less than five total infections in prison environments that are enforcing varying interventions; values calculated from stochastic computations of in-prison disease model

Mitigation measures	Frequency that the introduction of a new infection amongst prisoners will create an outbreak of 5 or fewer cases (%)
Hospitalisation and isolation, no reduction in force of infection	54.5
Hospitalisation and isolation, 25% reduction in force of infection	69.8
Hospitalisation and isolation, 50% reduction in force of infection	86.8
Hospitalisation and isolation, 75% reduction in force of infection	98.3

**Table 4:** Calculated probabilities that an incoming infected prisoner will remain undetected following spending a given amount of time reverse cohorting, while being subjected to tests of given sensitivity

	Testing sensitivity					
RC period	0.5	0.6	0.7	0.8	0.9	1
None	0.6990	0.6380	0.5780	0.5180	0.4570	0.3970
5	0.3991	0.3151	0.2423	0.1754	0.1313	0.0940
7	0.3702	0.2813	0.2029	0.1393	0.0849	0.0432
10	0.3538	0.2638	0.1822	0.1128	0.0557	0.0134
14	0.3488	0.2574	0.1754	0.1058	0.0485	0.0031

## Appendix. In-prison outbreak model description

The disease model that we have elected to use to simulate disease dynamics in a prison setting is based upon an Susceptible – Exposed – Exposed – Infectious – Infectious – Recovered (SEEIIR) compartmental model. The SEEIIR model was chosen as our basis due to it providing the best fit to COVID-19 CHESS and death data. However, this model (presented below) captures the behaviour of an outbreak occurring in an open population with no mitigating efforts being enforced:

However, following analyses of case data from the Diamond Princess, it was inferred that a sizeable proportion of infections were going undetected (due to being asymptomatic) while continuing to spread infection [1], [2]. As such, we sought to account for this behaviour in our model by allowing cases to progress into “infectious” states that will either be detected at some point during their infection, subsequently being removed from the prison population, or will remain undetected before eventually recovering (while remaining in the prison population). The main difference between these two “infectious” states is that the will-be-detected states have a much higher rate of removal (under the assumption that detected infections will be removed from the population soon after incubation has completed, and symptoms become observable) and will thus drive further infections for a shorter period. Note that in the main work, we refer to these individuals as the “clinically attacked” population. Whereas those in the will-not-be-detected states will be able to propagate the disease until they fully recover. The probability of detection is determined by the parameter k_c_. We also wished for our model to be able to treat both mild and severe infections. To this end, we incorporated two pathways that will-be-detected cases might progress towards recovery; being either medically isolated (representing the inmate having a mild infection) or hospitalised (representing the inmate suffering a severe infection). Probability of hospitalisation (and hence severe infection) after detection is described by the parameter k_h_. We present this model below:

**In-prison disease model parameterisations and assumptions**

Our disease model has been parametrised according to the following:

- In the unmitigated case, prisoners recover on average after 4 days (t_g_ = 4.0). We have then chosen to set *β* = 0.75 to achieve an R_0_ value of 3.0 (to be in line with early approximations of the reproductive number at the height of the UK’s first wave [3])
- Infected prisoners take on average 6 days to incubate (t_a_ = 6.0)
- A prison contains 678 prisoners; obtained by averaging prison data as reported in August 2020 [4]
- There is a 50% probability that infected prisoners shall be clinically attacked (k_c_ = 0.5)
- 5.8% of infected prisoners will be hospitalised (k_h_ = 0.058)
- It takes on average 1 day for clinically attacked prisoners to be detected (t_d_ = 1.0)
- It takes on average 7 days for clinically attacked inmates to fully recovery if suffering from mild symptoms (t_m_ = 7.0), and an average 22 days for clinically attacked inmates that have been hospitalised to fully recover (t_h_ = 22.0)
- 1.2% of the prison population are highly vulnerable
- Prison populations are uniform (no account for age, health or other differences) and mix homogeneously. There is no mixing with non-prison population, and staff are not considered within this model
- There is no birth or death process such as prisoners arriving or leaving
- Transition times between compartments follow Erlang distribution (two compartments for E, I_u_, I_d_, M and H)
- Individuals who are hospitalised do not return to the general prison population.

**Our model has also been based upon the following assumptions:**

1. For each introduction of the disease to prison this modelling assumes only a single index case:
  - When the reproduction number is greater than 1, this seeding number will not be significantly affected by additional infections.
  - Once there is transmission within a prison, this will outweigh any risk of importing an infection until the epidemic has been brought under control or reached its natural end.
  - Multiple outbreaks are not considered.
  - Should a significant number of susceptible individuals remain within an institution’s population following an outbreak, which is likely when intervention measures are in place, then subsequent introductions could lead to additional outbreaks.
  - Similarly, following an outbreak in an institution that has a high population turnover it is highly likely that the susceptible population may be sufficiently replenished to permit secondary outbreaks even where a substantial outbreak has already occurred.
2. Not all disease introductions will result in an epidemic due to stochastic die-out. As the initial number of infections increases, the chance of stochastic die-out occurring decreases. We only consider scenarios where more than 5 infections are observed.
3. Figures, unless specified, are generated from the average prison population of 678.

**Reverse cohorting model description**

To calculate the expected probabilities that infected prisoners will be detected at each testing point of the considered scenarios (defined by unique combination of testing sensitivity and time spent in the reverse cohorting unit, RCU), we have used a Monte Carlo based model that incorporates known aspects of COVID-19’s epidemiology. This model simulates prisoners being infected at some random time, -t_0_ (which we assume to have occurred at any point during the 14 days prior to entering the reverse cohorting process; this time is sampled from a uniform distribution across this period), before attempting to enter the RCU at time t = 0. Each prisoner is also assigned an incubation time, T_inc_ (randomly sampled according to the distribution described below), after which they will be detectable by testing.

After initialisation, each simulated prisoner then attempts to enter the prison through the RCU, where the model proceeds as described:

- If T_inc_ < t_0_, we assume the infected prisoner has become detectable prior to entering the RCU and will thus detectable to the test administered prior to entry. We then use a random process (with a probability of success equal to that scenarios testing sensitivity) to decide whether that person is detected or not. If detected, the prisoner is then recorded as “*non-entry*”.
- If T_inc_ > t_0_ or testing failed to detect symptoms, the prisoner proceeds to enter reverse cohorting where they then spend the next *n* days.
- If T_inc_ < t_0_ + *n*, we assume the infected prisoner has become detectable during the reverse cohorting process. The same random process as above is again used to determine whether the prisoners will be detected by the test administered prior to exit from the RCU. If detection is successful, the prisoner is removed from the model, being recorded as “*detected*”.
- Else, if T_inc_ > t_0_ + *n* or testing failed to detect symptoms, that prisoner is deemed to have been undetected, and will in theory be released into the prison population, being recorded as *“undetected”*.
- This process is then repeated 100,000 times for each testing sensitivity and cohorting period being considered. For each run, we record the number of simulated individuals that are detected prior to entering reverse cohorting, detected after reverse cohorting and those that are not detected.
- We then take ratio of each of these values against the total number of starting prisoners; this gives us an approximation to the probability that any infected prisoner will be detected by either test or make it undetected into the wider prison population under each scenario.

It is important to remember that this model only considers infected prisoners (every simulated individual is assumed to have already been infected by the time they attempt to enter the RCU) and therefore, the final values outputted by this model represent the proportion of the incoming prisoners that “survive” (remain undetected) the reverse cohorting process given that that have already been infected.

**Reverse cohorting model assumptions:**

- All simulated prisoners have, at some point prior to entering the RCU, been infected with COVID-19
- Incubation time is sampled according to a log-normal distribution with the parameters
- *µ* = 1.6112, *σ* = 0.47238
- For each prisoner, the time of infection is sampled according to uniform distribution over the 14 days prior to entering the RCU
- This model does not consider disease recovery
- Prisoners are only tested upon entry into the RCU, and once they have spent the specified amount of time in the RCU (if they proceed so far)
- If a prisoner has become detectable prior to a test, a weighted coin-toss (with probability of success equal to the testing sensitivity) is used to decide whether testing correctly detects them
- All prisoners undergo the reverse cohorting process
- There is no disease transmission at any stage of the model
- Simulated individuals proceed through the model one at a time, and thus enter self-isolation alone
- Disease prevalence remains constant throughout each considered scenario

**Bibliography**

[1] Emery, J.C. *et al.* (2020), “The contribution of asymptomatic SARS-CoV-2 infections to transmission on the Diamond Princess cruise ship”, *Elife*, Vol. 9, Aug, doi: 10.7554/eLife.58699.

[2] Buitrago-Garcia, D. *et al.* (2020), “Occurrence and transmission potential of asymptomatic and presymptomatic SARS-CoV-2 infections: a living systematic review and meta-analysis”, *PLoS Med.*, Vol. 17, No. 9, p. e1003346, Sep, doi: 10.1371/journal.pmed.1003346.

[3] Mahase, E. (2020), “Covid-19: What is the R number?”, *BMJ*, Vol. 369, p. m1891, May, doi: 10.1136/bmj.m1891.

[4] Prison population figures (2020), “GOV.UK.”, [Online], available at: www.gov.uk/government/statistics/prison-population-figures-2020 (accessed 13 August 2020).

Figure A1

Figure A2
